# Preparation and evaluation of the immune efficacy of an inactivated fowl adenovirus 8a serotype oil emulsion vaccine

**DOI:** 10.1016/j.heliyon.2024.e26578

**Published:** 2024-02-18

**Authors:** Jingqi Wu, Xiao Lu, Lingling Song, Liping Liu, Yuehua Gao, Hongmei Li, Kexiang Yu, Lihong Qi

**Affiliations:** a Institute of Animal Science and Veterinary Medicine, Shandong Academy of Agricultural Sciences, Jinan, China; b Poultry Institute, Shandong Academy of Agricultural Sciences, Shandong Provincial Key Laboratory of Poultry Diseases Diagnosis and Immunology, Jinan, China; c Shandong Provincial Key Laboratory of Animal Biotechnology and Disease Control and Prevention, Shandong Agricultural University, Tai’an, 271018, China; d College of Veterinary Medicine, Shandong Agricultural University, Tai’an, 271018, China; e Shandong Provincial Engineering Technology Research Center of Animal Disease Control and Prevention, Shandong Agricultural University, Tai’an, 271018, China

**Keywords:** Fowl adenovirus, FAdV-8a, Inclusion body hepatitis, Oil emulsion inactivated vaccine

## Abstract

In recent years, fowl adenovirus (FAdV) transmission has significantly increased worldwide, leading to substantial economic losses in the poultry industry. The virus causes hepatitis-hydropericardium syndrome (HHS) and inclusion body hepatitis (IBH). The prevalent FAdV strains in China are FAdV-4, FAdV-8a, FAdV-8b, and FAdV-11. Vaccines for FAdV-4 and FAdV-8b, which prevent HHS and IBH, are available commercially, but no vaccine exists for FAdV-8a. To address this issue, we developed a vaccine using an oil emulsion to inactivate the FAdV-8a serotype. Additionally, we built a fluorescence quantitative PCR for the detection of the virus. The lowest concentration detected was 4.11 × 10^1^ copies/μL. The study's results illustrated that the FAdV-8a oil emulsion vaccine effectively produced significant antibodies and offered ample protection for poultry. This vaccine can potentially limit the transmission of IBH resulting from FAdV-8a in China.

## 1 Introduction

In March 2022, the International Committee on Taxonomy of Viruses (ICTV) divided the Adenoviridae family in their latest virus classification report (https://ictv.global/taxonomy). Adenoviridae was divided into six genera [1]: *Mastadenovirus, Aviadenovirus, Atadenovirus, Siadenovirus, Ichtadenovirus*, and *Testadenovirus*. The fowl adenovirus is placed in the genus *Aviadenovirus*, which comprises 16 species, including *Aviadenovirus leucophthalmi, Duck aviadenovirus B, Fowl aviadenovirus A-E, Goose aviadenovirus A, Pigeon aviadenovirus A, Pigeon aviadenovirus B, Turkey aviadenovirus B-D, Psittacine aviadenovirus B, Psittacine aviadenovirus C, and Falcon aviadenovirus A. Fowl adenovirus* (FAdV) is a nonenveloped double-stranded DNA virus [2] that can infect chickens, leading to variable degrees of hepatitis-hydropericardium syndrome (HHS) and inclusion body hepatitis (IBH) [3], as well as reduced egg production [4].

FAdV is responsible for widespread sickness and significant economic losses [5], and it can be transmitted vertically [6] through the yolk sac, chick embryo chorioallantoic membrane, and allantoic cavity, as well as horizontally via contaminated feed, water, or contact [7]. Vertical transmission is considered the primary spread method, and the virus replicates primarily in chickens' digestive and respiratory tracts [8]. The disease can be prevalent year-round, with a peak incidence during the summer and rainy season. IBH outbreaks have increased in the past two decades, with the virus responsible for its global spread [9]. IBH primarily affects broiler chickens under 7 weeks of age [10], while layer hen infections have been reported occasionally. Because IBH-infected chickens often have concurrent disorders, some of the characteristics of field outbreaks are difficult to replicate in the laboratory. Among the 12 serotypes of FAdV, the most common serotypes of IBH in chickens are FAdV D and FAdV E [11]. FAdV-4, FAdV-8a, and FAdV-8b are the predominant serotypes in China. IBH is characterized by a large, swollen, friable liver with a marbled subepithelial reticulum of fine linear and stellate hemorrhages [2]; pale, bloated, mottled kidneys with a hemorrhagic renal cortex; and in some instances, an inflated, hemorrhagic spleen [12]. Inclusion bodies are present, and hepatocytes develop steatosis [13].

Vaccination is presently the primary technique for disease prevention and control [14], although cross-protection between FAdV serotypes is limited [15], and vaccine development is lacking. Inactivated vaccines are cheaper to produce, have a higher safety profile, and are easier to store and transport than other vaccines [16]. Inactivated vaccines have been used on a larger scale against FadV than other types of vaccines [17].

Inactivated vaccines are a type of vaccine that uses chemical or physical methods to inactivate the pathogen during preparation. Compared to other types of vaccines, inactivated vaccines are less costly to produce and have the advantage of being safer and easier to store and transport. Viruses are killed during the preparation of inactivated vaccines and lose their biological activity. These viruses retain their original structure and antigenicity but can no longer replicate or cause infection. Inactivated vaccines are, therefore, effective in inducing the body's immune system to produce specific antibodies, thereby preventing the onset and spread of disease. Inactivated vaccines are more accessible to apply than other types of vaccines, and they do not require special storage conditions or cold chain transport, making them more easily distributed around the globe, especially in areas where medical resources are scarce, or infrastructure is inadequate [18].

A highly effective inactivated oil emulsion vaccine has been successfully developed in the present study using FAdV-8a. The virus was isolated from the livers of FAdV-infected chickens. Test results showed that the vaccine elicited a robust immune response in SPF chickens after immunization. The vaccine also demonstrated excellent immunoprotective efficacy, ultimately inhibiting the cloacal shedding phenomenon, and showed no safety concerns. This vaccine is a promising candidate strain for developing FAdV-8a vaccines with broad market application.

## 2 Materials and methods

### 2.1 Virus and cell line

The FAdV-8a strain CY21 used in this study was taken from a broiler farm in Shandong Province. The farm was experiencing a disease called inclusion body hepatitis (IBH), and the Poultry Disease Laboratory obtained the isolate at the Shandong Academy of Agricultural Sciences. The chicken Leghorn male hepatoma (LMH) cell line (ATCC #CRL-2117) was used for cell culture, and it was grown in Dulbecco's Modified Eagle Medium (DMEM) (Gibco, USA) supplemented with 10% fetal bovine serum (FBS) (PAN, Germany) and 1% penicillin-streptomycin. The cells were maintained at 37 °C in a humidified environment with 5% CO2.

### 2.2 Virus passage and titration

LMH cell lines were used to grow the virus in 25 cm2 cell culture flasks. When the cells reached about 80% confluence, the culture medium was removed, and the cells were washed with phosphate-buffered saline (PBS). Then, 200 μL of a 100-fold diluted virus solution was added and incubated for an hour. After that, 5 mL of maintenance medium containing 1% fetal bovine serum (FBS) was added, and the cytopathic effect was monitored daily. The CY21 strain was passaged continuously for 15 generations in LMH cells. Virulence values were measured for the 3rd, 6th, 9th, 12th, and 15th generations to create a virulence change curve. Also, generations with high virulence were inoculated into LMH cells, and samples were taken at 6, 12, 18, 24, 36, 48, 60, 72, 84, and 96 h after injection to produce a growth curve.

### 2.3 Preparation of oil-adjuvant inactivated FAdV-8a vaccine

The antigen was inactivated using a final concentration of 0.2% formaldehyde. It was then added to the culture medium obtained from LMH cells infected with FAdV-8a. The inactivated antigen solution was mixed with white oil (HengFa, Guangzhou, China) at a ratio of 1:3. This mixture was then centrifuged at 6000 rpm for 10 min in a high-speed centrifuge.

### 2.4 Fluorescence quantitative PCR

Fluorescence quantitative PCR was performed using a Roche LightCycler 96 Real-Time PCR System (Mannheim, Switzerland). The primers were meticulously designed to target the hexon gene region of FAdV-8a, with FAdV-Q1: 5′-TTTGCCATCAAGAATCTGCT-3′ and FAdV-Q2: 5′-CTGATTGCTGGTGTTGTGGT-3′. An 189-bp sequence was inserted into a pMD18-T vector, and 4.11 × 10^1^ to 4.11 × 10^6^ copies/μL were used as the PCR template to establish a standard curve. The specificity of the assay was assessed by testing a range of viruses, including FAdV-8a-CY21, FAdV-8b, FAdV-1, FAdV-4, FAdV-11, IBV, AIV-H9, CREOV, CIAV, NDV, and IBDV, with ultrapure water serving as the negative control. Sensitivity was established by testing six serial dilutions of recombinant plasmid DNA that ranged from 4.11 × 10^1^ to 4.11 × 10^6^ copies/μL. Recombinant plasmids containing 4.11 × 10^1^ to 4.11 × 10^6^ copies/μL were utilized to evaluate the intra- and intergroup reproducibility.

The Roche LightCycler 96 Real-Time PCR System (Mannheim, Switzerland) performed fluorescence quantitative PCR. Primers were designed to specifically target the hexon gene region of FAdV-8a, with FAdV-Q1: 5′-TTTGCCATCAAGAATCTGCT-3′) and FAdV-Q2: 5′-CTGATTGCTGGTGTTGTGGT-3′. An 189-bp sequence was inserted into a pMD18-T vector, and a standard curve was established using 4.11 × 10^1^ to 4.11 × 10^6^ copies/μL as the PCR template. The assay's specificity was tested using ultrapure water as the negative control and a range of viruses, including FAdV-8a-CY21, FAdV-8b, FAdV-1, FAdV-4, FAdV-11, IBV, AIV-H9, CREOV, CIAV, NDV, and IBDV. Sensitivity was evaluated by testing six serial dilutions of recombinant plasmid DNA that ranged from 4.11 × 10^1^ to 4.11 × 10^6^ copies/μL. Reproducibility within and between groups was assessed using recombinant plasmids containing 4.11 × 10^1^ to 4.11 × 10^6^ copies/μL.

### 2.5 Animal experiment

To evaluate the effectiveness of the inactivated FAdV-8a vaccine, 32 specific pathogen-free (SPF) chickens were used in the study. The chickens were 21 days old and kept in separate positive-pressure isolators. They were randomly divided into four groups, each containing eight chickens. Group A was given 0.3 mL of the vaccine containing 10^6.5^ TCID_50_/0.1 mL via intramuscular injection. Group B and Group C were given 0.3 mL of the vaccine containing 10^5.5^ TCID_50_/0.1 mL and 10^4.5^ TCID_50_/0.1 mL, respectively. Group D was given 0.3 mL of a mixture of DMEM and white oil emulsion at a 1:3 ratio and was used as the unvaccinated control.

On Day 21, after infection, blood samples were taken from all the chickens and tested with the BioChek FAdV-I antibody ELISA kit from BioChek (China). The viral challenge involved the administration of FAdV-8a-CY21 (10^6^ TCID_50_/bird) intramuscularly at a dose of 0.3 mL per chicken.

We collected cloacal swabs from five chickens in each group during the study at 3, 6, 9, 12, and 15 days after infection. The samples were placed in 2 mL centrifuge tubes, and 1 mL of saline was added to each tube. After shaking the tubes, they were subjected to two freeze-thaw cycles and centrifuged at 8000 rpm for 15 min. We collected the supernatant and used fluorescence quantitative PCR for shedding detection.

Three chickens from each group were examined 6 days post-infection (dpi) to observe organ lesions. The heart, liver, spleen, lung, kidney, brain, bursa, and duodenum were collected, weighed, and homogenized in a saline solution with a ratio of 1:10. The homogenate was subjected to two freeze-thaw cycles, followed by centrifugation at 8000 rpm for 15 min. The supernatant was collected for detection using fluorescence quantitative PCR.

### 2.6 Statistics

The statistics analyses of antibody and viral copy numbers were conducted using ANOVA by Prism 6 (GraphPad). Differences were considered to be significant at *p < 0.05, **p < 0.01, ***p < 0.001 or ****p < 0.0001.

## 3 Results

### 3.1 The viral load and growth curve of CY21

**Fig. 1:**
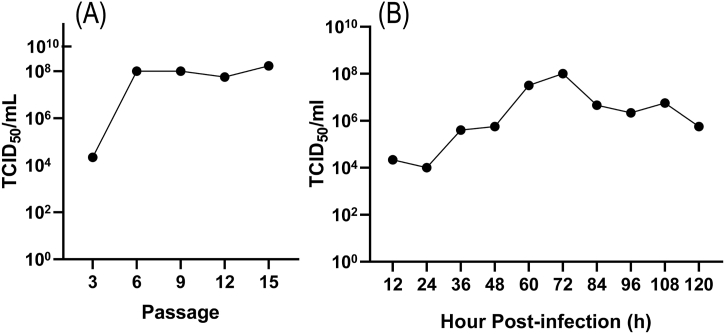
Growth curve of FAdV-8a and the change in the toxic value curve in LMH cells. (A) Changes in FAdV-8a virulence in successive passages of LMH cells. (B) Changes in FadV-8a virulence over time.

According to the Reed-Muench methodology, the viral TCID_50_ was calculated. CY21's titers remained at a TCID_50_ of 10^8.0^/mL after the 9th generation (Fig. 1A), and it reached its highest virulence of TCID_50_ 10^8.0^/mL 72 h after incubation (Fig. 1B).

### 3.2 Antibody responses of vaccinated chickens

**Fig. 2:**
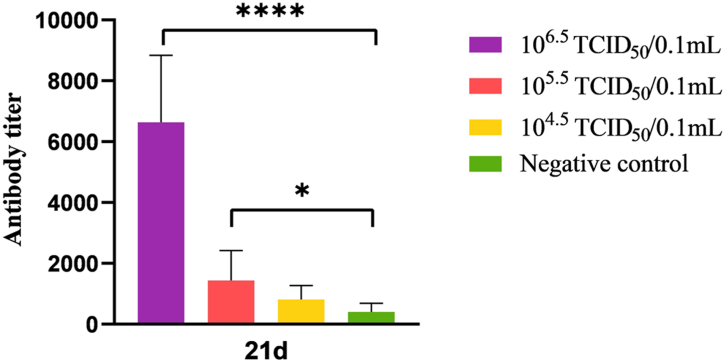
Antibody responses of vaccinated chickens (n = 8 birds for each construct). Group A was immunized with 10^6.5^ TCID_50_/0.1 mL. Group B was immunized with 10^5.5^ TCID_50_/0.1 mL. Group C was immunized with 10^4.5^/0.1 mL. Group D was the negative control.

The results of the study are presented in Fig. 2. It shows that all chickens in Group A had high levels of antibodies compared to the control group (p < 0.0001). In contrast, only half of the chickens in Group B had low levels of antibodies compared to the control group (p < 0.05). Out of all the chickens in Group C, only two showed positive results, while all chickens in Group D had negative results.

### 3.3 Clinical symptoms and gross pathology

**Fig. 3:**
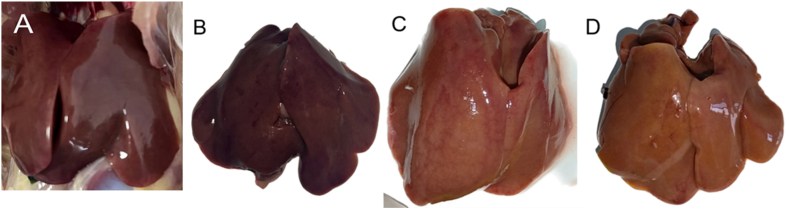
Clinical symptoms and gross pathology. (A) Immunized with 10^6.5^ TCID_50_/0.1 mL; no obvious liver lesions. (B) Immunized with 10^5.5^ TCID_50_/0.1 mL; no obvious liver lesions. (C) Immunized with 10^4.5^/0.1 mL, the liver was swollen, red, and yellow with hemorrhagic spots. (D) DMEM control group: liver is swollen with blunt rounded edges, overall earthy yellow color, hemorrhagic areas on the surface, friable.

During the experiment, the chickens in Groups A and B didn't show any clinical signs, while those in Groups C and D showed depression and decreased feeding at 3 days post-infection (dpi). However, none of the chickens died during the experiment. At 6 dpi, three chickens from each group underwent autopsy. Upon visual inspection, Groups A (Fig. 3A) and Groups B (Fig. 3B) showed no obvious lesion symptoms. On the other hand, the livers of chickens in Group C appeared swollen, turned pale yellow, and had red bleeding spots visible on the surface (Fig. 3C). The livers of chickens in Group D were also swollen with bluntly rounded edges, had bleeding spots on the surface, and became brittle and friable (Fig. 3D).

### 3.4 Establishment of a fluorescence quantitative PCR method

#### 3.4.1 Standard curves

After optimization, we established the reaction system and conditions for the fluorescence PCR assay. The reaction system was composed of 10 μL of TB Green Premix Ex, with a final concentration of 0.1 μM for both upstream and downstream primers, and 2 μL of DNA template, adjusted to a final volume of 20 μL with water.

The reaction conditions consisted of a predenaturation step of 30 s at 95 °C, followed by denaturation at 95 °C for 10 s, annealing at 58 °C for 15 s, extension at 72 °C for 15 s, and 40 cycles. During each extension cycle, fluorescence signals were collected at 72 °C.

**Fig. 4:**
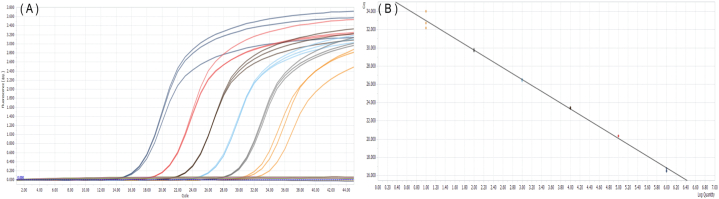
Amplification curve and standard curve analysis.

We generated standard curves using plasmid DNA with concentrations ranging from 4 × 10^6^ to 4 × 10^1^ copies/μL (Fig. 4A). The linear regression equation for the standard curve had a correlation coefficient of 0.998 and an amplification efficiency of 103.9% (Fig. 4B).

#### 3.4.2 Specificity

**Fig. 5:**
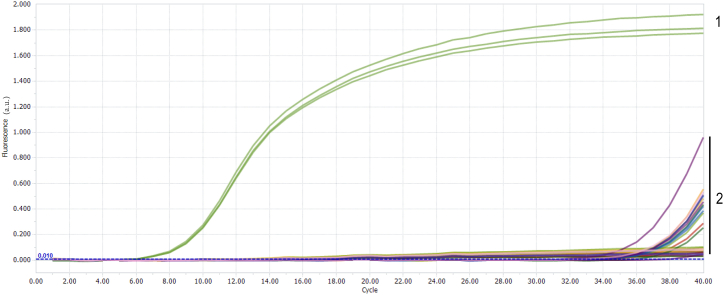
Specificity curve. Curve 1 corresponds to FAdV-8a. Curve 2 corresponds to other viruses and the negative control.

The assay showed high specificity, with only FAdV-8a-CY21 yielding positive results, while all other samples and the blank control were negative or had CT values exceeding 36.59, as shown in Fig. 5.

#### 3.4.3 Sensibility

**Fig. 6:**
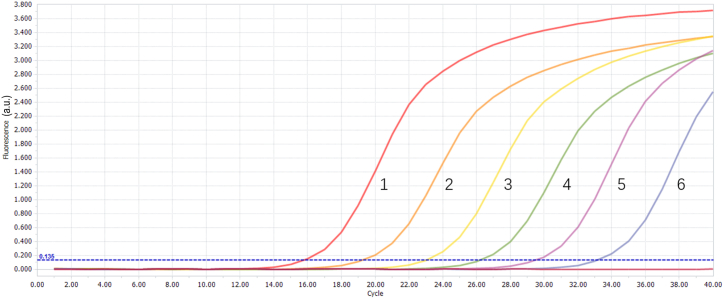
Sensitivity testing. Fluorescence quantitative PCR sensitivity. Curves 1–6 correspond to 4.11 × 10^6^∼4.11 × 10^1^ copies/μl of recombinant plasmid DNA.

Recombinant plasmids with a concentration ranging from 4.11 × 10^1^ to 4.11 × 10^5^ copies/μL at 10-fold dilution were used as templates for the assay. The fluorescence PCR method detected a minimum concentration of 4.11 × 10^1^ copies/μL, as shown in Fig. 6.

### 3.5 Detection of viral load in tissues

**Fig. 7:**
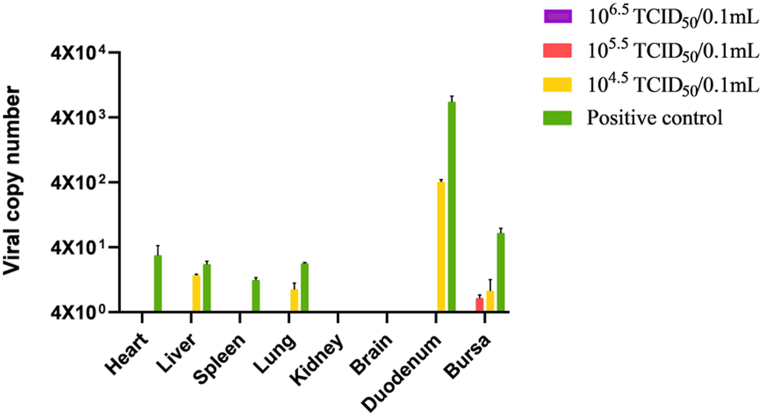
Viral titers in samples (heart, liver, spleen, lung, kidney, brain, duodenum, and bursa) from FAdV-8a-infected chickens(n = 3 birds for each construct). Group A was immunized with 10^6.5^ TCID_50_/0.1 mL. Group B was immunized with 10^5.5^ TCID_50_/0.1 mL. Group C was immunized with 10^4.5^ TCID_50_/0.1 mL. Group D was an unvaccinated control.

The results of the study are presented in Fig. 7. Viral DNA was not found in the kidney or brain. Group A showed no viral DNA in any organs, while Group B had trace amounts of viral DNA only in the bursa. Both Groups C and D exhibited viral DNA detection in the heart, liver, spleen, lung, duodenum, and bursa. The duodenum showed the highest peak with a viral copy number of 4 × 104.3 copies/μL. There was no viral DNA detected in the brain or kidney.

### 3.6 Detection of shedding

The results indicate that reducing the antigen titer to less than 10^4.5^ TCID_50_/0.1 mL (100-fold dilution group) does not prevent the shedding of the challenge test chickens. Similarly, an increase to 10^5.5^ TCID_50_/0.1 mL (10-fold dilution group) only partially inhibits shedding by 40%. However, when the antigen titer reaches 10^6.5^ TCID_50_/0.1 mL (in the primary fluid group), it completely suppresses the shedding of the challenge test chickens (Table 1). These findings suggest that the inactive CY21 vaccine is highly immunogenic, as it not only stimulates high levels of antibodies but also effectively inhibits shedding, demonstrating its robustness.

**Table 1:** Virus detection in each group after the challenge.

Group	3 dpi	6 dpi	9 dpi	12 dpi	15 dpi	21 dpi
10^6.5^ TCID_50_/0.1 mL	0/5	0/5	0/5	0/5	0/5	0/5
10^5.5^ TCID_50_/0.1 mL	0/5	2/5	3/5	1/5	0/5	0/5
10^4.5^ TCID_50_/0.1 mL	4/5	5/5	5/5	1/5	1/5	0/5
Positive control	5/5	5/5	5/5	4/5	3/5	0/5

## 4 Discussion

FAdV is widespread in vertebrates in nature, and each genus has its independent evolutionary lineage, presumably coevolving with its respective vertebrate hosts [[19], [20], [21]]. Recombination occurs in FAdV-D and FAdV-E, especially in FAdV-E [22], which accounts for many cases [23,24]. In Europe and North America [8], inclusion body hepatitis is the predominant form [7,[25], [26], [27], [28]], while in Asia, both IBH and HHS are common [29,30]. In China, four serotypes are prevalent: FAdV-4, FAdV-8a, FAdV-8b, and FAdV-11 [[31], [32], [33], [34], [35], [36]], among which virulent FAdV-8a is a less pathogenic virus. Although FAdV-8a has a low lethality rate, generally not exceeding 20% [8], the virus can lead to severe consequences such as immunosuppression, decreased production performance, and secondary infections and is currently more difficult to treat.

A study has shown that FAdV-8a and FAdV-11 offer partial cross-protection, while there is no cross-protection between FAdV-8b and FAdV-11 [37]. Therefore, developing a FAdV-8a vaccine can partially protect against FAdV-8b and FAdV-11 infections and has the potential for promising vaccine candidate vaccines.

In this investigation, we prepared a vaccine utilizing the virulent FAdV-8a CY21 strain, which was clinically isolated and inactivated via an oil emulsion. The serum obtained on Day 21 after a single immunization displayed a high immune antibody level. These results are consistent with the findings of P.A. Steer-Cope et al. [37], which demonstrated that the inactivated FAdV-8a vaccine elicited serum antibodies that neutralized FAdV-8a with titers ranging from 600 to 20,000 after immunization. Moreover, the cloacal shedding assay revealed that when the antigen titer reached 10^4.5^ TCID_50_/0.1 mL, it could not inhibit the shedding of challenge test chickens. These observations highlight that when the virulence of the immunogen got a certain threshold, the CY21 inactivated vaccine elicited a high level of antibody response, thereby effectively inhibiting the replication and transmission of the virus. Thus, the vaccine exhibited good immunogenicity and immunoprotective effects, thereby holding potential for developing FAdV-8a vaccine candidates. In addition, related studies suggest that the protective effects of the FAdV-8a virus may extend to serotype D [37]. Further research is needed to determine whether the inactivated vaccine of the CY21 strain prepared in this study has protective effects against other serotypes.

The focus of this study is the evaluation of antibody levels and vaccine protective efficacy after a single vaccine immunization using an inactivated FAdV-8a oil emulsion vaccine, which is the first of its kind in China. It is noteworthy that certain aspects of this study merit further exploration, including the applicability of the vaccine to other serotypes of the virus and its long-term sustainability in practical application. These areas require thorough investigation through additional studies to enhance this vaccine's theoretical foundation and efficacy.

## Data availability statement

The authors confirm that the data supporting the findings of this study are available within the article or its supplementary materials.

## Ethics statement

The Poultry Institute, Shandong Academy of Agricultural Sciences reviewed and approved the animal study (SAAS2022012).

## CRediT authorship contribution statement

**Jingqi Wu:** Writing – original draft, Validation, Methodology, Investigation. **Xiao Lu:** Validation, Methodology, Investigation, Data curation. **Lingling Song:** Supervision, Formal analysis, Conceptualization. **Liping Liu:** Resources, Project administration, Data curation. **Yuehua Gao:** Software, Investigation. **Hongmei Li:** Writing – review & editing, Validation, Supervision, Methodology, Funding acquisition. **Kexiang Yu:** Writing – review & editing, Supervision, Project administration, Methodology, Funding acquisition, Conceptualization. **Lihong Qi:** Writing – review & editing, Supervision, Resources, Funding acquisition, Conceptualization.

## Declaration of competing interest

The authors declare that they have no known competing financial interests or personal relationships that could have appeared to influence the work reported in this paper.
